# Estimation of cardiac amyloid infiltration with myocardial T1 mapping correlates with severity of cardiac involvement

**DOI:** 10.1186/1532-429X-15-S1-P161

**Published:** 2013-01-30

**Authors:** Eduardo Pozo, Jose M Castellano, Anubhav Kanwar, Rajiv Deochand, Sarayu Ramachandran, Claudia Calcagno, Pablo Pazos, Ines Garcia-Lunar, Matthew D Cham, Valentin Fuster, Javier Sanz

**Affiliations:** 1 Cardiology, Mount Sinai School of Medicine, New York City, NY, USA

## Background

Post-contrast myocardial T1-mapping with cardiac magnetic resonance (CMR) has shown usefulness in the diagnosis of cardiac amyloid. A lower value indicates increase in extracellular volume and thereby amyloid deposition.

The aim of this study is to assess the association between the amount of amyloid deposition as estimated by the degree of post-contrast myocardial T1 reduction and the severity of left ventricular (LV) involvement in cardiac amyloidosis.

## Methods

Consecutive patients referred for 3.0 Tesla CMR with a final diagnosis of cardiac amyloidosis (defined as positive cardiac biopsy and/or typical diffuse, predominantly subendocardial pattern of delayed contrast enhancement) were retrospectively evaluated. The septal E/E' ratio as an index of LV filling pressures was measured with doppler echocardiography (n=18, 69%). Indexed LV volumes and mass, LV ejection fraction, and LV basal anteroseptal and inferolateral wall thicknesses were determined from cine CMR. Hemodynamic data from cardiac catheterization and brain natriuretic peptide (BNP) serum levels were available in 9 (36%) and 14 (54%) patients. respectively. Myocardial, endocardial, blood, and skeletal muscle were quantified after Gd-DTPA administration on a previously validated Look-Locker sequence using dedicated software. Correlations between myocardial T1 values and other indices of cardiac involvement were performed using Pearson correlation coefficients.

## Results

We included 26 patients (17 males [65.4%], age 68±13 years) with a final diagnosis of cardiac amyloid. There was an inverse correlation between myocardial/blood T1 ratio and basal anteroseptal (r=-0.519, p=0.008) and inferolateral (r=-0.498, p=0.011) wall thickness. Similarly endocardial/blood T1 ratio was inversely correlated with basal anteroseptal (r=-0.595, p=0.002; Figure) and inferolateral (r=-0.429, p=0.032) wall thickness. No significant associations were noted with LV volumes, ejection fraction, or mass. Otherwise, myocardial and endocardial T1 time showed an inverse correlation with BNP (r=-0.659, p=0.014 and r=-0.610, p=0.027 respectiveley). In addition, myocardial/skeletal muscle T1 ratio showed a significant inverse association with pulmonary capillary wedge pressure (r=-0.724, p=0.042).

**Figure 1:**
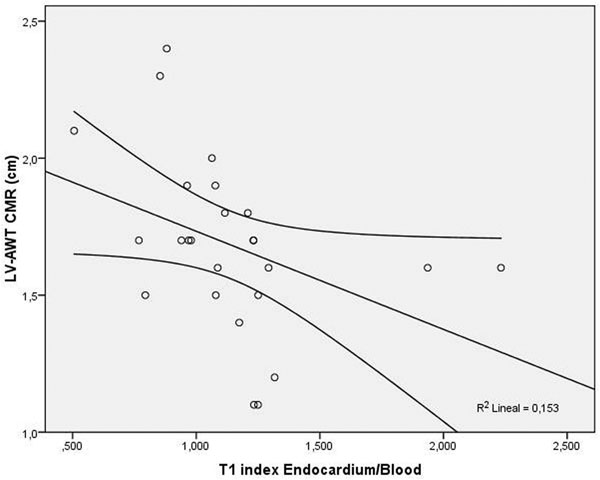
Correlation between LV anterior wall thickness (LV-AWT) and the T1 time index of endocardium and blood.

## Conclusions

The degree of postcontrast myocardial T1 reduction is associated with markers of disease severity in cardiac amyloidosis, suggesting a potential role of T1-mapping for noninvasive quantification of cardiac amyloid deposition with CMR.

## Funding

No funding sources have to be declared.

